# New Inventions

**Published:** 1898-03

**Authors:** 


### NEW INVENTIONS.

A bridle-blind of metal.

A horseshoeing apparatus consisting of a framework with lever attachments by which the fore and hind limbs may be maintained in position for adjusting shoe.

A horseshoe with recessed toe and heels for the adjusting of calks.

A design of a horseshoe-calk to be bolted to heels of shoe.

A bridle-bit with two bars with looped ends, these connected with interlocking loops for straps for check-rein.

A tubular hitching-post, with chain fastened to inner part of post.

A shipping-box for animals, with an enlarged bottom and tapering sides to lid, with slats.

An improved animal-shears.

A channelled rubber-tread horseshoe, prongs along channel to retain the rubber.

A sheet-metal harness-hame.

A bottle-washing machine.

An equalizer device for regulating the gait of horses and preventing them from leaving their feet, by a frame over the shoulder connected with cords to boots on the limbs.

An animal-holding apparatus, with pivotal table and means for securing the limbs.

A horseless carriage.

A horseshoe with continuous calk on inner edge, a bead on the outer edge, with groove for placing and directing nails, which are flush with beaded edge.

---

The Pennsylvania State Board of Veterinary Medical Examiners will probably hold a special meeting of the Board during March to conduct an examination of several applicants.

